# Multi-level learning: improving the prediction of protein, domain and residue interactions by allowing information flow between levels

**DOI:** 10.1186/1471-2105-10-241

**Published:** 2009-08-05

**Authors:** Kevin Y Yip, Philip M Kim, Drew McDermott, Mark Gerstein

**Affiliations:** 1 Department of Computer Science, Yale University, 51 Prospect Street, New Haven, CT 06511, USA; 2 Terrence Donnelly Centre for Cellular and Biomolecular Research, 6/F, 160 College Street, University of Toronto, Toronto, Ontario M5S 3E1, Canada; 3 Program in Computational Biology and Bioinformatics, Yale University, New Haven, CT 06520, USA; 4 Department of Molecular Biophysics and Biochemistry, Yale University, 266 Whitney Avenue, New Haven, CT 06520, USA

## Abstract

**Background:**

Proteins interact through specific binding interfaces that contain many residues in domains. Protein interactions thus occur on three different levels of a concept hierarchy: whole-proteins, domains, and residues. Each level offers a distinct and complementary set of features for computationally predicting interactions, including functional genomic features of whole proteins, evolutionary features of domain families and physical-chemical features of individual residues. The predictions at each level could benefit from using the features at all three levels. However, it is not trivial as the features are provided at different granularity.

**Results:**

To link up the predictions at the three levels, we propose a multi-level machine-learning framework that allows for explicit information flow between the levels. We demonstrate, using representative yeast interaction networks, that our algorithm is able to utilize complementary feature sets to make more accurate predictions at the three levels than when the three problems are approached independently. To facilitate application of our multi-level learning framework, we discuss three key aspects of multi-level learning and the corresponding design choices that we have made in the implementation of a concrete learning algorithm. 1) Architecture of information flow: we show the greater flexibility of bidirectional flow over independent levels and unidirectional flow; 2) Coupling mechanism of the different levels: We show how this can be accomplished via augmenting the training sets at each level, and discuss the prevention of error propagation between different levels by means of soft coupling; 3) Sparseness of data: We show that the multi-level framework compounds data sparsity issues, and discuss how this can be dealt with by building local models in information-rich parts of the data. Our proof-of-concept learning algorithm demonstrates the advantage of combining levels, and opens up opportunities for further research.

**Availability:**

The software and a readme file can be downloaded at . The programs are written in Java, and can be run on any platform with Java 1.4 or higher and Apache Ant 1.7.0 or higher installed. The software can be used without a license.

## Background

The functions of many proteins depend highly on their interactions with other proteins. Complete protein-protein interaction (PPI) networks provide insights into the working mechanisms of proteins at a global level. While high-throughput experiments such as yeast two-hybrid (Y2H) [1-4] and tandem-affinity purification with mass spectrometry (TAP-MS) [5,6] have enabled the survey of whole PPI networks, the resulting data are noisy with a lot of false positives and false negatives [7,8].

The construction of more reliable PPI networks has been assisted by computational techniques. These techniques usually employ a supervised [9-12] or unsupervised and topological [13-16] machine learning method to predict the interaction of proteins. While some of the methods could predict PPI networks with high accuracy, they do not explain how the proteins interact. For instance, if protein A interacts with both proteins B and C, whether B and C could interact with A simultaneously remains unknown, as they may or may not compete for the same binding interface of A. This observation has led to the recent interest in refining PPI networks by structural information about domains [17-19]. It has also called for the prediction of protein interactions at finer granularities.

**Figure 1:**
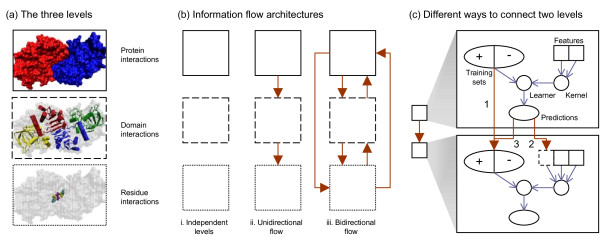
**Schematic illustration of multi-level learning concepts**. (a) The three levels of interactions. Top: the PDB structure 1piw of the homo-dime r yeast. NADP-dependent alcohol dehydrogenase 6. Middle: each chain contains two conserved Pfam domain instances, PF00107 (inner) and PF08240 (outer). The interaction interface is at PF00107. Bottom: two pairs of residues predicted by iPfam to interact: 283 (yellow) with 287 (cyan), and 285 (purple) with 285. (b) The three information flow architectures. i: independent levels, ii: unidirectional flow (illustrated by download flow), iii: bidirectional flow. (c) Coupling mechanisms for passing information from one level to another. 1: passing training information to expand the training set of the next level, 2: passing predictions as an additional feature of the next level, 3: passing predictions to expand the training set of the next level.

Since binding interfaces of proteins are enriched in conserved domains in permanent interactions [20], it is possible to construct a second-level interaction network with protein interactions annotated by the corresponding domain interactions. An even finer third-level interaction network involves the residues mediating the interactions (Figure 1, visualization of (a) by VMD [21]).

As will be described in the next section, some recent studies have started to perform interaction predictions at the domain and residue levels. The data features used by each level are quite distinct. While protein level features are mostly from functional genomic and proteomic data such as gene expression and sub-cellular localization of whole genes and proteins, domain level features are mainly evolutionary information such as phylogenetic-occurrence statistics of the domain families, and residue level features are largely structural or physical-chemical information derived from the primary sequences.

In the literature of domain-level prediction, the term "domain" is usually used to mean a domain family, which could have multiple occurrences in different proteins. In this study we use the terms "domain family" and "domain instance" to refer to these two concepts respectively, in order to make a clear distinction between them. For example, PF07974 is a domain family from Pfam, where ADP1_YEAST.PF07974 is a domain instance in the protein ADP1_YEAST.

Since the data features of the three levels describe very different aspects of the biological objects, potentially they could contribute to the prediction of different portions of the interaction networks. For example, some protein interactions could be difficult to detect using whole-protein level features since they lack fine-grained physical-chemical information. These can be supplemented by the residue level features such as charge complementarity.

Likewise, for the protein interactions that occur within protein complexes, there could be a high correlation between the expressions of the corresponding genes. With proper gene expression datasets included in the protein features, there is a good chance of correctly predicting such protein interactions. Then if one such interaction involves a pair of proteins each with only one conserved domain, it is very likely that the domain instances actually interact.

One may worry that if the predictions at a particular level are inaccurate, the errors would be propagated to the other levels and worsen their predictions. As we will discuss, this issue can be handled algorithmically by carefully deciding what information to propagate and how it is propagated. With a properly designed algorithm, combining the predictions and utilizing the data features of all three levels can improve the predictions at each level.

In this work we propose a new multi-level machine-learning framework that combines the predictions at different levels. Since the framework is also potentially useful for other problems in computational biology that involve a hierarchy, such as biomedical text mining (a journal contains papers and a paper contains key terms), we start with a high-level description of multi-level learning and discuss three key aspects of it. Then we suggest a practical algorithm for the problem of predicting interactions at the protein, domain and residue levels, which integrates the information of all three levels to improve the overall accuracy. We demonstrate the power of this algorithm by showing the improvements it brings to the prediction of yeast interactions relative to the predictions from independent levels (Software available [additional file 1]).

### Related work

Two main ingredients of protein-protein interaction predictions are the selection of a suitable set of data features, and an appropriate way to integrate them into a learning method. Many kinds of features have been considered [12], including sub-cellular localization [22], gene expression [23,24], and phylogenetic profiles [25]. With the many different kinds of data features, Bayesian approaches [10] and kernel methods [9,12,26] are natural choices for integrating them into a single learning algorithm. The former unifies the whole inference process by a probabilistic framework, while the latter encodes different kinds of data into kernel matrices that can be combined by various means [27].

Domain family-domain family interaction predictions are related to the more general goal of identifying protein interaction interfaces. While some studies tackle the problem using features at the domain level only [28], most other work assumes that a set of protein-protein interactions are known a priori, and the goal is to predict either domain family interactions (i.e., which domain families have their instances interact in at least one pair of proteins) or domain-instance interactions (i.e., through which domain instances do proteins interact in known interactions) [28-49]. The data features are mainly derived from statistics related to the parent proteins. For example, for a pair of domain families, the frequency of co-occurrence in interacting proteins is an informative feature, since a higher frequency may indicate a larger chance for them to be involved in mediating the interactions.

At a finer level, identifying protein interaction interfaces involves the prediction of residue interactions, which could be divided into two sub-tasks: 1) predicting which residues are in any interaction interfaces of a protein [50], and 2) predicting which of these interfaces interact [51]. Data features are mainly derived from the primary protein sequences or from crystal structures if they are assumed available. Docking algorithms [52] represent related approaches, but have a fundamentally different focus: Their goal is to utilize largely physical information to deduce the structure of the complex from the unbound protein structures, a considerably harder problem. Therefore, we do not consider them in this article and focus on large-scale techniques.

From a theoretical perspective, our multi-level learning framework is loosely related to co-training [53] and the meta-learning technique stacking [54]. We will compare them with our framework after introducing the information flow architectures and the coupling mechanisms in Sections and respectively. Also, our framework by nature facilitates semi-supervised learning [55]. We will briefly discuss semi-supervised learning and its relationships with PSI-BLAST [56] in Section.

### Problem definition

We now formally describe the learning problem we tackle in this study. The inputs of the problem consist of the following:

• Objects: a set of proteins, each containing the instances of one or more conserved domains, each of which contains some residues. Each protein, domain instance and residue is described by a vector of feature values. Some additional features are available for pairs of objects, such as the likelihood for a pair of proteins to interact according to a high-throughput experiment.

• Gold standard positive sets of known protein-protein, domain instance-domain instance and residue-residue interactions. As in other studies on protein interaction networks, we use the term "gold standard set" to mean a set of sufficiently reliable data useful for the prediction purpose, instead of a ground-truth set that is absolutely correct. The positive sets could be 1) contaminated with false positives, and 2) incomplete, with false negatives, and a pair of upper-level objects in the positive set may not have any corresponding lower-level object pairs known to be in the positive sets.

• Gold standard negative sets of non-interactions at the three levels.

We assume no crystal structures are available except for the proteins in the gold-standard positive sets, so that the input features cannot be derived from known structures. This is a reasonable assumption given the small number of known structures as compared to the availability of other data features.

The objective is to use the gold standard sets and the data features to predict whether the object pairs outside the gold standard sets interact or not. Prediction accuracies are estimated by cross-validation using holdout testing examples in the gold standard sets not involved in the training process.

In this study we focus on kernel methods [57] for learning from examples and making predictions. The main goal of this study is to explain how the predictions at the different levels can be integrated, and to demonstrate the resulting improvements in accuracy. We do not attempt to boost the accuracy at each individual level to the limit. It may be possible to improve our predictions by using other features, learning algorithms, and parameter values. As we will see, the design of our algorithm provides the flexibility for plugging in other state-of-the-art learning methods at each level. We expect that the more accurate the individual algorithms are, the more benefits they will bring to the overall accuracy through the multi-level framework.

## Methods

In order to develop a method for predicting interactions at all three levels in a cohesive manner, we need to define the relationships between the levels, which is the topic of Section. We first describe two information flow architectures already considered in previous studies, and then propose a new architecture that maximally utilizes the available data. In Section we discuss various possible approaches to coupling the levels, i.e., ways to pass information between levels. In Section we discuss the data sparsity issue. In particular, we describe the idea of local modeling, which is also useful for network predictions in general. Finally, in Section we outline the actual concrete algorithm that we have developed and used in our experiments.

### Information flow architectures

#### Architecture 1: independent levels

A traditional machine-learning algorithm learns patterns from one single set of training examples and predicts the class labels of one single set of testing instances. When there are three sets of examples and instances instead, the most straightforward way to learn from all three levels is to handle them separately and make independent predictions (Figure 1bi). We use this architecture to setup the baseline for evaluating the performance of the other two architectures.

#### Architecture 2: unidirectional flow

A second architecture is to allow downward (from protein to domain to residue) or upward (from residue to domain to protein) flow of information, but not both (Figure 1bii). This architecture is similar to some previous domain-level interaction methods described above, which also use information from the protein level. However, in our case protein interactions are not assumed to be known with certainty. So only the training set and the predictions made from the training set at the protein level can be used to assist the domain and residue levels.

#### Architecture 3: bidirectional flow

A third architecture is to allow the learning algorithm of each level to access the information of any other levels, upper or lower (Figure 1biii). By allowing both upward and downward flow of information, this new architecture is the most flexible among the three, and is the architecture that we explore in this study. Theoretically, this architecture is loosely related to co-training [53], which assumes the presence of two independent sets of features, and each is capable of predicting the class labels of a subset of data instances. Here we have three sets of features, each of which is capable of predicting a portion of the whole interaction network. Practical extensions to the ideal co-training model allow partially dependent feature sets and noisy training examples, which fit our current problem. Learning proceeds by iteratively building a classifier from one feature set, and adding the highly confident predictions as if they were gold-standard examples to train another classifier using the other feature set. The major difference between our bidirectional-flow architecture and co-training is the presence of a hierarchy between the levels in our case, so that each set of features makes predictions at a different granularity.

### Different approaches to coupling the levels

To design a concrete learning algorithm, we need to specify what information is to be passed between different levels and how it is passed. Here we suggest several possibilities, and briefly discuss the pros and cons of each of them.

### What information to pass

#### i. Training data

One simple idea is to pass training data to other levels (Figure 1c, arrow 1). This can be useful in filling in the missing information at other levels. For example, many known protein interactions do not have the corresponding 3D structures available, so there is no information regarding which domain instances are involved in the interactions. The known protein interactions can be used to compute statistics for helping the prediction of domain-level interactions.

#### ii. Training data and predictions

The major limitation of passing only training data is that the usually much larger set of data instances not in the training sets (the "unlabeled data") would not benefit from multi-level learning. In contrast, if the predictions made at a level are also passed to the other levels, much more data instances could benefit (Figure 1c, arrow 2 and 3). For instance, if two domain instances are not originally known to interact, but they are predicted to interact by the domain-level features with high confidence, this information directly implies the interaction of their parent proteins.

Algorithms adopting this idea are semi-supervised in nature [55], since they train on not only gold-standard examples, but also predictions of data instances that are originally unlabeled in the input data set. Note that the idea of semi-supervised learning has been explored in the bioinformatics literature. For instance, in the PSI-BLAST method [56], sequences that are highly similar to the query input are iteratively added as seeds to retrieve other relevant sequences. These added sequences can be viewed as unlabeled data, as they are not specified in the original query input.

### How the information is passed

#### i. Combined optimization

To pass information between levels, a first approach is to combine the learning problems of the different levels into a single optimization problem. The objective function could involve the training accuracies and smoothness requirements of all three levels. This approach enjoys the benefits of being mathematically rigorous, and being backed by the well-established theories of optimization. Yet the different kinds of data features at the different levels, as well as noisy and incomplete training sets, make it difficult to define a good objective function. Another drawback is the tight coupling of the three levels, so that it is not easy to reuse existing state-of-the-art prediction algorithms for each level.

#### ii. Predictions as additional features

Another approach is to have a separate learning algorithm at each level, and use the predictions of a level as an additional feature of another level (Figure 1c, arrow 2). For example, if each pair of proteins is given a predicted probability of interaction, it can be used as the value of an additional feature 'parent proteins interacting' of the domain instance pairs and residue pairs. In this approach the different levels are loosely coupled, so that any suitable learners can be plugged into the three levels independently, and the coupling of the levels is controlled by a meta-algorithm.

A potential problem is the weighting of the additional features from other levels relative to the original ones. If the original set of features is large, adding one or two extra features without proper weighing would have negligible effects on the prediction process. Finding a suitable weight may require a costly external optimization or cross-validation procedure. For kernel methods, an additional challenge is integrating the predictions from other levels into the kernel matrix, which could be difficult as its positive semi-definiteness has to be conserved.

The idea of having a meta-algorithm that utilizes the predictions of various learners is also used in stacked generalization, or stacking [54]. It treats the predictions of multiple learners as a new set of features, and uses a meta-learner to learn from these predictions. However, in our setting, the additional features come from other levels instead of the same level.

#### iii. Predictions as augmented training examples

A similar approach is to add the predictions of a level to the training set of another level (Figure 1c, arrow 3). The resulting training set involves the original input training instances and augmented training data from other levels, with a coefficient reflecting how much these augmented training data are to be trusted according to the training accuracy of the supplying level. This approach also has the three levels loosely coupled.

A potential problem of this training set expansion approach is the propagation of errors to other levels. The key to addressing this issue is to perform soft coupling, i.e., to associate confidence values to predictions, and propagate only highly confident predictions to other levels. For kernel methods, this means ignoring objects falling in or close to the margin. This approach is similar to PSI-BLAST mentioned above, which selectively includes only the most similar sequences in the retrieval process.

In this study, we focus on this third approach. It requires a learning method for each level, while the control of information flow between the different levels by means of training set expansion forms the meta-algorithm. Since each level involves only one set of features and one set of data instances, traditional machine learning methods can be used. We chose support vector regression (SVR) [58], which is a type of kernel method. We used regression instead of the more popular support vector machine classifiers [59] because the former can accept confidence values of augmented training examples as inputs, and produce real numbers as output, which can be converted back into probabilities that reflect the confidence of interactions.

### Global vs. local modeling, and data sparsity issues

#### Global modeling

Taking a closer look at the prediction problem at each individual level, one would realize that applying a traditional learning method is actually non-trivial since we are dealing with network data. In a traditional setting, each training instance has a class label and the job of a learning algorithm is to identify patterns in the feature values for predicting the class label of each unlabeled object. In our current situation, each data instance is a pair of biological objects (proteins/domain instances/residues), with two possible class labels: interacting and non-interacting. In order to construct a learner, one would need features for pairs of objects. A model can then be learned using a traditional machine learning method for all object pairs. We call this 'global modeling' since a single regression model is built for all the data instances. Global modeling has a number of major drawbacks:

1. Features for object pairs: it is not easy to construct features for pairs of objects, since most available data features are for single objects. This is particularly a problem for kernel methods, which require a kernel matrix to encapsulate the similarity between each pair of data instances. For network data, this means a similarity value for each pair of object pairs. While methods have been proposed to construct such kernel matrices [9], the resulting kernels, while formally correct, are difficult to interpret.

2. Time complexity: working with pairs of objects squares the time requirement with respect to the number of objects in the dataset. While state-of-the-art implementations of kernel methods could easily handle thousands of proteins, it would still be challenging to deal with millions of protein pairs, let alone the even more daunting numbers of domain instance pairs and residue pairs.

3. Space complexity: the kernel matrix has a size quadratic in the number of data instances. With *n *objects at a level, there are *O*(*n*^2^) pairs and thus the kernel matrix contains *O*(*n*^4^) entries.

4. Sub-clusters: the two classes of data instances may contain many sub-clusters that cannot be handled by one single global model. For instance, proteins involved in permanent complexes may use a very different interaction mechanism from transient interactions in signaling pathways.

#### Local modeling

To avoid these problems, one alternative is local modeling [26]. Instead of building one single global model for all object pairs, one local model is built for each object. For example, if the dataset contains *n *proteins, then *n *models are built, one for each protein, for predicting whether this protein interacts with each of the *n *proteins. The advantages of local modeling are obvious: 1) data features are needed for individual objects only, 2) the time complexity is smaller than global modeling whenever the learning method has a super-linear time complexity, 3) much less memory space is needed for the kernel matrix, and 4) each object can have its very specific local model. For all these benefits, in our experiments we only considered local modeling.

Local modeling is most useful when the training data are abundant and evenly distributed across different objects, such that each object receives a reasonable amount of positive and negative examples to train its local model. However, when the training data are sparse and uneven, some objects may have insufficient (or none at all) training examples. For instance, among the millions of yeast protein pairs, there are only a few thousand known interactions, so many proteins have very few of them.

Our proposed solution uses concepts related to semi-supervised learning: use high confidence predictions to augment training sets. Suppose protein A has sufficient known positive and negative examples in the original training sets, and the local model learned from these examples predicts with high confidence protein B to be an interaction partner with A. Then when building the local model for B, A can be used as a positive training example. Predicted non-interactions can be added as negative examples in a similar way. This idea is consistent with the training set expansion method proposed above for inter-level communication. As a result, the information flow both between levels and within a level can be handled in a unified framework. The expanded training set of a level thus involves the input training data, highly confident predictions of the local models of the level, and highly confident predictions from other levels. Practically, training set expansion within the same level requires an ordered construction of the local models. Objects with many (input or derived) training examples should have their local models constructed first, as more accurate models are likely to be obtained from larger training sets. As these objects are added as training examples of their predicted interaction partners and non-partners, they would progressively accumulate training examples for their own local models.

#### The concrete algorithm

We now explain how we used the ideas described in the previous sections, namely bidirectional information flow, coupling by predictions passing, and local modeling with training set expansion, to develop out concrete learning algorithm for prediction of protein, domain instance and residue interactions. We first give a high-level overview of the algorithm, then explain the components in more detail.

The main steps of the algorithm are:

1. Setup a learning sequence of the levels.

2. Use the model learned for the first level in the sequence to predict interactions at the level.

3. Propagate the most confident predictions to the next level in the sequence as auxiliary training examples.

4. Repeat the previous two steps for the second and third levels, and so on.

#### Learning at each level

We use training set expansion with support vector regression (SVR) to perform learning at each level. Each pair of objects in the positive and negative training sets is given a class label of 1 and 0, respectively. An SVR model is learned for the object (e.g. protein) with the largest number of training examples (denoted as A). The model predicts a real value for each object, indicating the likelihood that it interacts with A. The ones with the largest and smallest predicted values are treated as the most confident positive and negative predictions, and are used to expand the training set. For example, if B is an object with the largest predicted value, then A and B are predicted to interact, and A is added as an auxiliary positive training example of B. After training set expansion, the next object with the largest number of training examples is re-determined, its SVR is learned, and the most confident predictions are used to expand the training set in the same manner. The whole process then repeats until all models have been learned. Finally, each pair of objects A and B received two predicted values, one from the model learned for A and one from the model learned for B. The two values are weighted according to the training accuracies of the local models for A and B to produce the predicted value for the pair. Sorting the predicted values in descending order gives a list of predictions from the pair most likely to interact to the one least likely. The list is then used to evaluate the accuracy by the area under the receiver operator characteristic curve (AUC) [60]. We have tried a range of values for defining the most confident set of predictions (results available at supplementary web site), and the general trends of prediction accuracies were observed to remain largely unchanged.

#### Setting up the learning sequence

One way to setup the learning sequence is to use the above procedure to deduce the training accuracy of the three levels when treated independently, then order the three levels into a learning sequence according to their accuracies. For example, if the protein level gives the highest accuracy, followed by the domain level, and then the residue level, the sequence would be "PDRPDR...", where P, D and R stand for the protein, domain and residue levels, respectively. Having the level with the highest training accuracy earlier in the sequence ensures the reliability of the initial predictions of the whole multi-level learning process, which is important since all latter levels depend on them. Notice that after learning at the last level, we feedback the predictions to the first level to start a new iteration of learning.

In our computational experiments we also tested the accuracy when only two levels are involved. In such situations, we simply bypassed the left-out level. For example, to test how much the domain and residue levels could help each other without the protein level, the learning sequence would be "DRDR...".

#### Propagating predictions between levels

The mechanism of propagating predictions from a level to another depends on the direction of information flow.

For an upward propagation (R→D, R→P or D→P), each object pair in the next level receives a predicted likelihood of interaction from each pair of their child objects. For example, if predictions are propagated from the domain level to the protein level, each pair of domain instances provides a predicted value to their pair of parent proteins. We tried two methods to integrate these values. In the first method, we normalize the predicted values to the [0, 1] range as a proxy of the probability of interaction, then use the noisy-OR function [48] to infer the chance that the parent objects interact. Let *X *and *Y *be the two sets of lower-level objects, and p(x, y) denotes the probability of interaction between two objects *x *∈ *X *and *y *∈ *Y*, then the chance that the two parent objects interact is 1 - ∏_*x*∈*X*, *y*∈*Y*_(1 - *p*(*x*, *y*)), i.e., the parent objects interact if and only if at least one pair of its children objects interact. In the second method, we simply take the maximum of the values. In the ideal case where all predicted values are either 0 or 1, both methods are exactly the same as taking the OR of the values. When the values are noisy, the former is more robust as it does not depend on a single value. Yet its value is dominantly affected by a large number of fuzzy predicted values with intermediate confidence, and is thus less sensitive. Since in our tests it does not provide superior performance, in the following we report results for the second method.

For a downward propagation (P→D, P→R or D→R), we inherit the predicted value of the parent pair as the prior belief that the object pairs from the two parents will interact. For example, if we are propagating information from the protein level to the domain level, each pair of domain instances has a prior belief of interaction equal to the predicted likelihood that their parent proteins interact.

In both cases, after computing the probability of interaction for each pair of objects in the next level based on the predicted values at the current level, we again add the most confident positive and negative predictions as auxiliary training examples for the next level, with the probabilities used as the confidence values of these examples.

In the actual implementation, we used the Java package libsvm [61] for SVR, and the Java version of lapack  for some matrix manipulations.

## Results

We tested the effectiveness of multi-level learning by predicting protein, domain instance and residue interactions of the yeast Saccharomyces cerevisiae.

### Data

#### Protein level

Data features were gathered from multiple sources (Table 1), including phylogenetic profiles [62], sub-cellular localization [22], gene expression [24,63], and yeast two-hybrid [1,4] and TAP-MS [5,6] networks. Each of them was turned into a kernel matrix and the final kernel was the summation of them, as in previous studies [12,26].

**Table 1:** Data features at the protein level.

Feature	Feature of	Data type	Kernel
COG (version 7) phylogenetic profiles [62]	Proteins	Binary vectors	Linear

Sub-cellular localization [22]	Proteins	Binary vectors	Linear

Cell cycle gene expression [24]	Proteins	Real vectors	Correlation (linear after standardization)

Environment response gene expression [63]	Proteins	Real vectors	Correlation (linear after standardization)

Yeast two-hybrid [1,4]	Protein pairs	Unweighted graph	Diffusion (*β *= 1)

TAP-MS [5,6]	Protein pairs	Weighted graph	Diffusion (*β *= 1)

A gold standard positive set was constructed from the union of experimentally verified or structurally determined protein interactions from MIPS [64], DIP [65] and iPfam [66] with duplicates removed. The MIPS portion was based on the 18 May 2006 version, and only physical interactions not obtained from high throughput experiments were included. The DIP portion was based on the 7 Oct 2007 version, and only interactions from small-scale experiments or multiple experiments were included. The iPfam portion was based on version 21 of Pfam [67]. A total of 1681 proteins with all data features and at least one interaction were included in the final dataset, forming 3201 interactions. A gold standard negative set with the same number of protein pairs was then created from random pairs of proteins not known to interact in the positive set [9,32].

#### Domain level

We included two types of features at the domain level: co-evolution and statistics related to parent proteins (Table 2). These are similar to the features used by previous studies for domain family/domain instance interaction predictions [28,47,68].

**Table 2:** Data features at the domain level.

Feature	Feature of	Data type	Kernel
Phylogenetic tree correlations [68] of Pfam alignments	Domain family pairs	Real matrix	Empirical kernel map [70]

In all species, number of proteins containing an in stance of the domain family	Domain families	Integers	Polynomial (d = 3)

In all species, number of proteins containing domain instances only from the family	Domain families	Integers	Polynomial (d = 3)

Number of domain instances of parent protein	Domain instances	Integers	Polynomial (d = 3)

Fraction of non-yeast interacting protein pairs contain ing instances of the two domains respectively are mediated by the domain instances*	Domain family pairs	Real matrix	Constant shift embedding [71]

Fraction of protein pairs containing instances of the two domains respectively are known to be interacting in the PPI training set*	Domain family pairs	Real matrix	Constant shift embedding

*: These two features were used with the unidirectional and bidirectional flow architectures only since they involve information about the training set of the protein level.

The gold standard positive set was taken from iPfam, where two domain instances are defined as interacting if they are close enough in 3D structure and some of their residues are predicted to form bonding according to their distances and chemistry. After intersecting with the proteins considered at the protein level, a total of 422 domain instance interactions were included, which involves 272 protein interactions and 317 domain instances from 223 proteins and 252 domain families. A negative set with the same number of domain instance pairs was then formed from random pairs of domain instances in the positive set. All known yeast Pfam domain instances of the proteins were involved in the learning, many of which do not have any known interactions in the gold standard positive set. Altogether 2389 domain instances from 1681 proteins and 1184 domain families were included.

#### Residue level

We used three data features derived from sequences (Table 3). Charge complementarity and other features likely useful for interaction predictions are implicit in the sequence profiles. The features are similar to those used in a previous study [51]. However, as we do not assume the availability of crystal structures of unlabeled objects, the secondary structures and solvent accessible surface areas we used were algorithmically predicted from sequence instead of derived from structures. We used SABLE [69] to make such predictions. Information about empirical kernel map and constant shift embedding can be found in [70] and [71], respectively.

**Table 3:** Data features at the residue level.

Feature	Feature of	Data type	Kernel
PSI-BLAST profiles	Residues and neighbors	Vectors of real vectors	Summation of linear

Predicted secondary structures	Residues and neighbors	Vectors of real vectors	Summation of linear

Predicted solvent accessible surface areas	Residues and neighbors	Vectors of real numbers	Summation of circular

In a previous study [51], the feature set of a residue involves not only the features of the residue itself, but also neighboring residues closest to it in the crystal structure, which allows for the possibility that some of them are involved in the same binding site and thus have dependent interactions. In the absence of crystal structures, we instead included a window of residues right before and after a residue in the primary sequence to construct its feature set. We chose a small window size of 5 to make sure that the included residues are physically close in the unknown 3D structures.

The gold standard positive set was taken from iPfam, where the interacting residues are determined based on their proximity in known crystal structures of interacting proteins. Since there is a large number of residue pairs, we only sampled 2000 interactions, which involve 228 protein pairs, 327 domain instance pairs and 3053 residues from 195 proteins, 279 domain instances and 224 domain families. Only these 3053 residues were included in the data set. A negative set was created by randomly sampling from these residues 2000 residue pairs that do not have known interactions in iPfam.

#### Evaluation procedure

We used ten-fold cross validation to evaluate the performance of our algorithm. Since the objects in the three levels are correlated, an obvious performance gain would be obtained if in a certain fold the training set of a level contains some direct information about the testing set instances of another level. For example, if a residue interaction in the positive training set comes from a protein pair in the testing set, then the corresponding protein interaction can be directly inferred and thus the residue interaction would create a fake improvement for the predictions at the protein level. This problem was avoided by partitioning the object pairs in the three levels consistently. First, the known protein interactions in iPfam were divided into ten folds. Then, each domain instance interaction and each residue interaction was put into the fold in which the parent protein interaction was assigned. Finally, the remaining protein interactions and all the negative sets were randomly divided into ten folds.

Each time, one of the folds was held out as the testing set and the other nine folds were used for training. We used the area under the ROC (Receiver Operator Characteristics) curve (AUC) [60] to evaluate the prediction accuracies. For each level, all object pairs in the gold standard positive and negative sets were sorted in descending order of the predicted values of interaction they received when taking the role of testing instances. The possible values of AUC range from 0 to 1, where 1 corresponds to the ideal situation where all positive examples are given a higher predicted value than all negative examples, and 0.5 is the expected value of a random ordering.

We compared the prediction accuracies in three cases: independent levels, unidirectional flow of training information only, and bidirectional flow of both training information and predictions. For the latter two cases, we compared the performance when different combinations of the three levels were involved in training.

For independent levels, we trained each level independently using its own training set, and then used the predictions as initial estimates to retrain for ten feedback iterations. This iterative procedure was to make sure that any accuracy improvements observed in the other architectures were at least in part due to the communications between the different levels, instead of merely the effect of semi-supervised learning at a single level. For unidirectional flow, we focused on downward flow of information. The levels were always arranged with upper levels coming before lower levels.

## Results

Table 4 summarizes the prediction accuracies of the three levels. All numbers correspond to the average results among the ten feedback iterations. Each row represents the results of one level. For unidirectional flow and bidirectional flow, the levels involved in training are also listed. For example, the PR column of unidirectional flow involves the use of the protein-level training sets in setting up the initial estimate of the residue interactions. This has no effect on the predictions at the protein level since information only flows downward. The cell at the row for protein interactions is therefore left blank. The best result in each row is in bold face.

**Table 4:** Prediction accuracies (AUC) of the three levels with different information flow architectures and training levels.

	Independent levels	Unidirectional flow			Bidirectional flow			
Level		PD	PR	DR	PD	PR	DR	PDR

Proteins	0.7153				0.7205	0.7227		**0.7257**

Domains	0.5214	0.5854			**0.7015**		0.6796	0.6986

Residues	0.5675		0.5296	0.5128		0.6581	0.6182	**0.7361**

We first notice that the results for independent levels are consistent with our expectations. Having many diverse data features, the protein level has a satisfactory accuracy. On the other hand, the accuracies of the domain and residue levels were relatively low due to their weak and noisy features. Note that we are predicting whether two arbitrary domain instances or two arbitrary residues interact, rather than only those in known interacting protein pairs. This setting is more realistic for organisms with no known protein interaction network, and the problem is significantly harder than when the protein interaction network is available.

**Figure 2:**
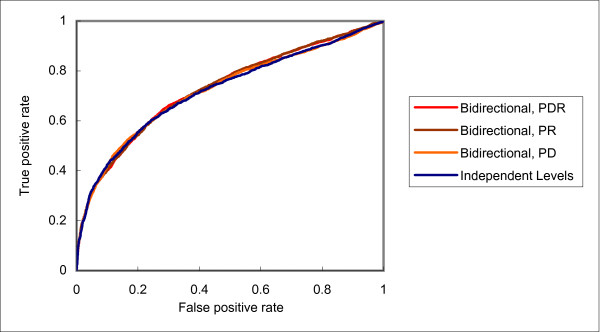
**Receiver operator characteristic (ROC) curves of protein interaction predictions with different frameworks and training levels**.

**Figure 3:**
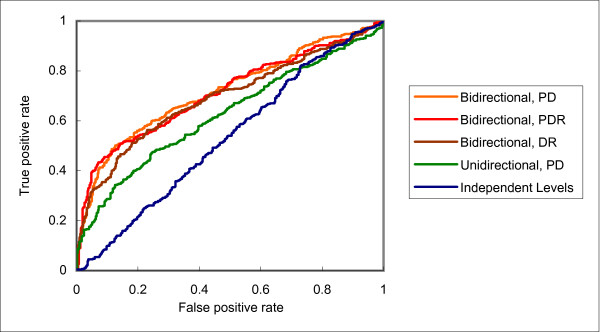
**Receiver operator characteristic (ROC) curves of domain interaction predictions with different frameworks and training levels**.

**Figure 4:**
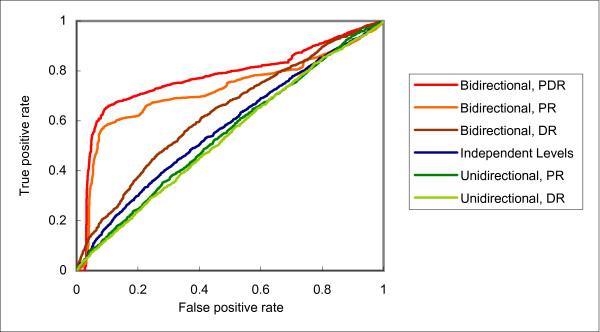
**Receiver operator characteristic (ROC) curves of residue interaction predictions with different frameworks and training levels**.

Downward flow of training information did help the prediction of domain instance interactions. However, the results of the residue level are quite unsatisfactory, with accuracies even lower than those with independent levels no matter assisted by the training examples of the protein level or domain level. In contrast, the results for bidirectional flow are encouraging. In all cases, the accuracies are higher than the other two architectures. For example, while using the domain level to help the residue level decreased the accuracy of the latter from 0.5675 to 0.5128 with unidirectional flow, the accuracy was increased to 0.6182 with bidirectional flow. As an illustration of the difference in performance of the three architectures, the various ROC curves of protein, domain and residue interaction predictions are shown in Figures 2, 3 and 4, respectively.

The improvements for both the domain and residue levels are quite dramatic, with maximum AUC gains of more than 0.15. This clearly shows the benefits of passing not only training information, but also highly confident predictions. Consider a domain instance pair in the testing set of a certain fold. Since the corresponding parent protein pair must not be in the training set at the protein level of the fold, the passing of training information does not directly help predict the interaction status of the domain instance pair. On the other hand, if the interaction status of the protein pair is predicted correctly with high confidence, passing this information to the domain level can make a direct influence on the prediction of the domain instance interaction. For instance, if the protein pair is correctly predicted as not interacting, the domain instance pair would probably be correctly predicted as not interacting, too.

In general, it is observed that levels with a higher raw accuracy with independent levels could offer a bigger improvement to the other levels. For example, the protein level increased the accuracy of the residue interaction predictions from 0.5675 to 0.6581, while the domain level could only increased it to 0.6182. However, it is also crucial to note that although the domain level has a low accuracy with independent levels, it could still make good improvements to the prediction of residue interactions. This observation supports our design of passing only highly confident predictions in avoiding the propagation of errors. The combination of all three levels has the potential to further improve accuracy. For both the protein and residue levels, the best results were obtained when all three levels were involved in training. In particular, while each of the protein and domain levels improved the residue level by a certain amount, the combination of them provided yet another significant amount of improvement.

As a concrete example of information flow between the three levels, several of the interacting residue pairs between the hexokinase 1 (PF00349) and hexokinase 2 (PF03727) domains of the hexokinase isoenzyme 2 (HXK2) protein are correctly identified with high likelihood of interaction. For example, residue 46 in the hexokinase 1 domain and residue 278 in the hexokinase 2 domain are predicted to interact with a score of 0.89 out of 1.0. This might be partially due to the charge complementarity of the two residues in their PSI-BLAST profiles, with the most conserved residues being the positively-charged arginine and the negatively-charged aspartic acid at the two positions, respectively. The detecting of such residue interaction helps raise the likelihood of the corresponding domain interaction from a score of 0.27 to 0.72. In turn, this helps detect the self-interaction of the glucokinase (GLK1) protein, which also has the two domains. The interaction was verified in a two-hybrid assay [1].

## Discussion

The experimental results have demonstrated the great potential of linking up the prediction problems at the different levels. This initial success encourages deeper investigations of the idea along various directions. Algorithmically, other approaches to combining the different levels, including combined optimization and predictions as extra features, need to be studied. Currently the features at the domain and residue levels are weak, as reflected by their low accuracy when learned independently, and the small improvement they could cause to the protein level. It is interesting to study ways to improve the predictions at these two levels, and more directly extract the complementary information hidden in these levels that are useful for the protein level.

The current study is limited by a highly disproportionate dataset, with much more training examples at the protein level than the domain level. Together with a much weaker feature set, the raw accuracy at the domain level is much lower than the protein level, and the former could only slightly improve the predictions of the latter in the multi-level learning framework. It is hoped that as more structures of protein complexes are solved, the disproportionality would be alleviated. In the meantime, it is interesting to study ways to derive other features that could better predict the domain interactions, and the mechanism by which the domain-level information improved the protein-level predictions.

In this article we have pointed out some important issues of both multi-level learning and network prediction, including data sparseness and the existence of sub-clusters. While we have proposed methods to tackle them, a detailed analysis of how these issues affect prediction performance and what other algorithmic strategies for tackling them are yet to be studied.

To predict the whole interaction network, it is needed to reduce the time and space requirements. One possible way is to intelligently select what data to exclude, such as residues that are predicted to be buried deep inside the core of a protein. Another idea is to group objects into interaction groups so that each cluster can be handled independently.

More insights could be gained by studying some theoretical aspects of multi-level learning, such as the hierarchical structure of the prediction problems, and the issue of noisy and incomplete training sets. With multiple levels, performance evaluation is very tricky. As we discussed, careless definitions of training and testing sets could produce biases to the resulting performance. It is instrumental to study the optimal way of evaluation.

Biologically, there are many interesting follow-up questions to be studied. The intricate interactions between the different levels are not yet clear, and could form a larger study of how the predictions change after receiving information from the other levels. One could compare different kinds of data features at the three levels and identify the ones with the greatest complementary effects. Another direction is to choose different kinds of residue samples (e.g., only charged residues) and inspect the relative improvements they provide to the protein and domain levels, to determine the residues that are more significant in a protein interaction.

## Conclusion

In this article we have introduced the approach of integrating protein interaction prediction at the protein, domain and residue levels. We have described the potential benefits of this multi-level learning framework due to the availability of distinct and complementary data features at each level. We have defined three information flow architectures for learning from the different levels, and proposed various ways to couple the levels in terms of what and how information is passed between them. We have focused on the training set expansion method, which is a meta-algorithm that passes predictions of a level as augmented training examples of other levels. To avoid the propagation of errors, we have discussed soft coupling, which associates confidence values to predictions and passes only highly confident predictions other levels. The confidence values are used as the inputs of support vector regression. For learning the interaction network at each level, we have compared global and local modeling, which has also highlighted the issue of data sparsity. We have performed computational experiments using yeast data, and shown that the bidirectional flow of supervised and semi-supervised information between the different levels improved the predictions over independent levels and mere downward flow of training information. The evaluation procedure involved special experimental procedures including training-set balancing and consistent cross validation. Finally, we have suggested a number of follow-up research topics.

## Authors' contributions

All authors conceived the project and design. KY and PK prepared the data. KY implemented the algorithms, performed the computational experiments, and analyzed the results. KY and MG wrote the paper. All authors read and approved the document.

## Supplementary Material

Additional file 1 **The multi-level learning program**. Compiled Java code of the multi-level learning program. Click here for file
